# Construction of an economical xylose-utilizing *Saccharomyces cerevisiae* and its ethanol fermentation

**DOI:** 10.1093/femsyr/foae001

**Published:** 2024-01-24

**Authors:** Fan Li, Wenxin Bai, Yuan Zhang, Zijian Zhang, Deguo Zhang, Naidong Shen, Jingwei Yuan, Guomiao Zhao, Xiaoyan Wang

**Affiliations:** Nutrition and Health Research Institute, COFCO Corporation, No. 4 Road, South District, Beiqijia Town, Changping District, Beijing 102209, China; COFCO Biochemical and Bioenergy (Zhaodong) Co., Ltd., No. 24, Zhaolan Road, Zhaodong City, Suihua, Heilongjiang 151100, China; COFCO Corporation, COFCO Fortune Plaza, No.8, Chao Yang Men South St., Chao Yang District, Beijing 100020, China; Nutrition and Health Research Institute, COFCO Corporation, No. 4 Road, South District, Beiqijia Town, Changping District, Beijing 102209, China; Nutrition and Health Research Institute, COFCO Corporation, No. 4 Road, South District, Beiqijia Town, Changping District, Beijing 102209, China; COFCO Corporation, COFCO Fortune Plaza, No.8, Chao Yang Men South St., Chao Yang District, Beijing 100020, China; Nutrition and Health Research Institute, COFCO Corporation, No. 4 Road, South District, Beiqijia Town, Changping District, Beijing 102209, China; COFCO Corporation, COFCO Fortune Plaza, No.8, Chao Yang Men South St., Chao Yang District, Beijing 100020, China; COFCO Biotechnology Co., Ltd., No. 1, Zhongliang Avenue, Yuhui District, Bengbu, Anhui 233010, China; Nutrition and Health Research Institute, COFCO Corporation, No. 4 Road, South District, Beiqijia Town, Changping District, Beijing 102209, China; COFCO Corporation, COFCO Fortune Plaza, No.8, Chao Yang Men South St., Chao Yang District, Beijing 100020, China; COFCO Biochemical and Bioenergy (Zhaodong) Co., Ltd., No. 24, Zhaolan Road, Zhaodong City, Suihua, Heilongjiang 151100, China; COFCO Corporation, COFCO Fortune Plaza, No.8, Chao Yang Men South St., Chao Yang District, Beijing 100020, China; Nutrition and Health Research Institute, COFCO Corporation, No. 4 Road, South District, Beiqijia Town, Changping District, Beijing 102209, China; COFCO Corporation, COFCO Fortune Plaza, No.8, Chao Yang Men South St., Chao Yang District, Beijing 100020, China; Nutrition and Health Research Institute, COFCO Corporation, No. 4 Road, South District, Beiqijia Town, Changping District, Beijing 102209, China; COFCO Corporation, COFCO Fortune Plaza, No.8, Chao Yang Men South St., Chao Yang District, Beijing 100020, China

**Keywords:** *Saccharomyces cerevisiae*, industrial ethanol production, industrial material domestication, pilot-scale fermentation, transcriptome analysis

## Abstract

Traditional industrial *Saccharomyces cerevisiae* could not metabolize xylose due to the lack of a specific enzyme system for the reaction from xylose to xylulose. This study aims to metabolically remould industrial *S. cerevisiae* for the purpose of utilizing both glucose and xylose with high efficiency. Heterologous gene *xylA from Piromyces* and homologous genes related to xylose utilization were selected to construct expression cassettes and integrated into genome. The engineered strain was domesticated with industrial material under optimizing conditions subsequently to further improve xylose utilization rates. The resulting *S. cerevisiae* strain ABX0928-0630 exhibits a rapid growth rate and possesses near 100% xylose utilization efficiency to produce ethanol with industrial material. Pilot-scale fermentation indicated the predominant feature of ABX0928-0630 for industrial application, with ethanol yield of 0.48 g/g sugars after 48 hours and volumetric xylose consumption rate of 0.87 g/l/h during the first 24 hours. Transcriptome analysis during the modification and domestication process revealed a significant increase in the expression level of pathways associated with sugar metabolism and sugar sensing. Meanwhile, genes related to glycerol lipid metabolism exhibited a pattern of initial increase followed by a subsequent decrease, providing a valuable reference for the construction of efficient xylose-fermenting strains.

## Teaser

This research constructed a remarkable yeast strain able to utilize straws effectively to produce fuel ethanol. This strain shows promising prospects for industrial production better than other two commercial strains.

Abbreviations XI: Xylose isomerase
XR: Xylose reductase
XD: Xylitol dehydrogenase
XK: Xylulokinase
PPP: Pentose phosphate pathway
XU: Xylose utilization
SAC: Sugar–alcohol conversion
PCA: Principal component analysis
DEG: Differentially expressed genes
CCR effect: Effect of carbon decomposition
TCA cycle: Citrate cycle

## Highlights

- This research applies a combinatorial strategy with genetic editing and evolutionary engineering, overexpressed genes are integrated into genome for stable expression and lower burden. We finally acquired a strain with better performance of xylose utilization and stability under industrial environment compared with commercial strains.
- We tested the final strain in straw enzymatic hydrolysis liquid extrudate with 30 m^3^ pilot-scale fermentation, which verified the production advantage and production stability of this strain under real industrial environment.
- Comparison of the engineered strain with the commercial strains ABX-CIP1 and ABX-CIP2 reveals that the final engineered strain, ABX0928-0630, exhibits superior performance in terms of ethanol production yield and xylose consumption rate during mixed-sugar fermentation. Additionally, it produces lower concentrations of unwanted byproducts, such as xylitol and glycerol.
- Transcriptomic analysis reveals the increased efficiency of the final strain ABX0928-0630 in sugar uptake, the loss of glucose repression, and an enhanced ability for sugar sensing and ethanol response.

## Background

In order to reduce greenhouse gas emissions, clean energy sources such as fuel ethanol have been utilized as a substitution for a significant proportion of gasoline (Munack and Krahl 2010, Sandri et al. 2023). Compared with first-generation ethanol, which employs corn as a raw material, second-generation cellulose ethanol made from straw is cheaper and more environmentally friendly (Dos Santos et al. 2016a). Lignocellulosic raw materials such as corn stover are collected and pretreated to break down the lignin and cellulose firstly, then hydrolyzed through enzymes to release low-carbon sugars such as glucose and xylose, and fermented with industrial yeast strains to produce ethanol subsequently (Hahn-Hägerdal et al. 2007). The yeast strain *Saccharomyces cerevisiae* commonly utilized for first-generation ethanol production is firstly assessed for fermenting xylose but appears to lack the ability (Patiño et al. 2019, Stambuk 2019). Therefore, genetically engineered strains were constructed afterwards to enhance the metabolism of xylose (Sharma and Arora 2020).

**Figure 1.**
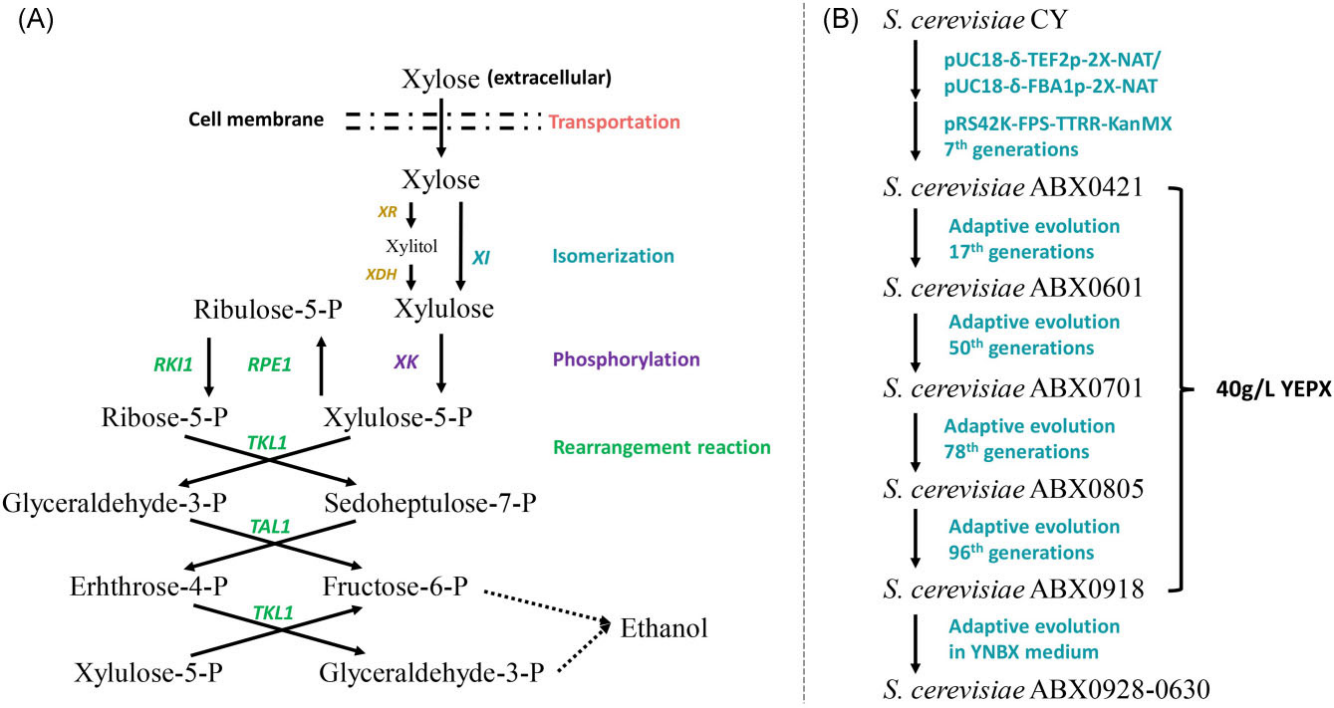
XI-driven xylose assimilation in engineered yeast. (A) Schematic illustration of the xylose assimilation module. (B) Schematic diagram of strain construction and domestication.

The main limitations of xylose metabolism in *S. cerevisiae* include xylose transportation, cofactor requirements of xylose-metabolizing enzymes and xylitol accumulation. To date, various strategies have been developed to modify the xylose metabolic pathway in *S. cerevisiae* (Kuyper et al. 2005a,b, Runquist et al. 2010, Sedlak and Ho 2004, Zhou et al. 2012). Generally, there are two pathways to transform xylose to xylulose in natural microorganisms, including a pathway via xylose isomerase (XI) and a pathway via xylose reductase (XR) and xylitol dehydrogenase (XD) (Fig. 1A). Engineered industrial *S. cerevisiae* strains that can effectively ferment and metabolize xylose to produce ethanol are constructed through heterologous expression of xylose metabolism pathway. XR and XD are endogenously expressed in *S. cerevisiae* while they are not as efficient due to redox imbalance. By changing the affinities of XR and XD for NADH and NADPH, it is possible to decrease xylitol accumulation and increase ethanol production (Almeida et al. 2011, Jo et al. 2016). In addition, a recent study proposed a strategy that involves integrating metabolic engineering with sugar sensing and signalomics in yeast strains to resolve the xylose paradox (Osiro et al. 2019). Compared with the redox pathway, the expression of xylose isomerization pathway would not cause coenzyme imbalance due to accumulation of intermediate xylitol, which is an ideal pathway for xylose metabolism (Brat et al. 2009, Zhou et al. 2012). After catalysis from xylose, xylulose is phosphorylated by endogenous xylulokinase (XK) and channelled into the pentose phosphate pathway (PPP) and glycolysis to produce ethanol (Van Maris et al. 2006).

Previous research has identified a number of processes as bottlenecks that restrict the xylose utilization (XU) efficiency, including the uptake of xylose (Runquist et al. 2009), the accumulation of xylitol (Zhu et al. 2021), the conversion of xylulose (Zhou et al. 2012), and the flux of the PPP pathway (Johansson and Hahn-Hägerdal 2002). One of the most straightforward strategies to diminish the bottlenecks is the overexpression of xylose utilizing related proteins (Kuyper et al. 2005a, Runquist et al. 2009). Increasing the copy number of XI encoding gene *xylA* has been identified as an important strategy to improve the xylose metabolism and ethanol production efficiency of yeast strains. After artificial domestication of recombinant bacteria with multiple copies of the gene in δ integration site, the gene copy number of the best two strains with xylose as the only carbon source increased to 36 and 26, respectively, while the control strain had no significant xylose consumption (Dos Santos et al. 2016b). In addition, studies showed that the consumption rate of xylose mutants obtained from the domestication of the recombinant strain was significantly increased, along with the ethanol yield of as high as 0.43 g/g xylose. The detection showed that the copy number and mRNA level of the *xylA* gene of the two domesticated strains were 8–32 times higher than those of the control strain (Zhou et al. 2012). The above studies indicated that the enzyme activity of XI was still the main limiting factor in XI pathway strains. XK encoded by gene *XKS1* is another necessary gene on the downstream metabolic pathway for both XR-XDH and XI pathways. Overexpression of *XKS1* gene can significantly reduce the accumulation of xylitol (Hohenschuh et al. 2015, Kim et al. 2013a). The activity of *XKS1* in wild-type *S. cerevisiae* is too low to meet the basic requirements of xylose fermentation for industrial transformation (Toivari et al. 2001). Moreover, compared with other yeasts, the metabolic flux of the nonoxidative phase PPP in *S. cerevisiae* is much lower (Öhgren et al. 2006). As the sole pathway for d-xylose metabolized to the central metabolism, PPP is critical for pentose metabolism and requires higher expression (Latimer and Dueber 2017). Overexpression of all PPP genes has been demonstrated to improve the xylsoe metabolism (Kobayashi et al. 2017) (Kobayashi et al. 2018). In addition to gene manipulation, mutagenesis, or adaptive domestication is also an essential step for increasing XU efficiency and obtaining stable expression hosts (Kuyper et al. 2005a, Shen et al. 2012, Zhou et al. 2012). Directed evolution techniques have been applied to sugar transporter proteins, enabling glucose/xylose cotransport and eliminating glucose inhibition in yeast (Li et al. 2016, Xu et al. 2020). Another study employed adaptive domestication to enhance the xylose consumption kinetics of an engineered strain under aerobic conditions, resulting in a 70% increase in xylose consumption rate (Thalita Peixoto et al. 2022).

Despite a number of studies achieving the production of cellulose by *S. cerevisiae*, these strains are still difficult to work in actual industrial production, mainly due to two reasons. The first one is the selection of hosts, most hosts selected are laboratory haploid strains, which are less robust than industrial diploid strains for fermentation (Karhumaa et al. 2005, 2007, Kuyper et al. 2005b). Another reason is the expression of proteins, most of them are expression based on plasmids, which are less stable than those integrated into genome (Meinander and Hahn‐Hägerdal 1997, Zhang et al. 1996). There have been attempts to integrate the XI into genome, but with a laboratory haploid strain (Tanino et al. 2010). Another research integrating seven different genes related to XU into genome was reported based on an industrial diploid strain (Demeke et al. 2013), while the XU efficiency in industrial material such as corn stover hydrolysate still needs improvement.

As mentioned earlier, the main challenge of ethanol production through xylose-fermenting lies in the utilization efficiency of industrial materials. However, effectively expressing XI in an industrial strain and testing it with real industrial production are rarely reported. To this end, the industrial diploid strain *S. cerevisiae* CY was chosen as the chassis host, involving a strategy that combines genetic engineering with adaptative evolution engineering. Genes related to XU were selected to be integrated into yeast chromosomes. Two expression cassettes including heterogenous XI gene Pi-*xylA* origin from *Piromyces*, and endogenous genes *XKS1, RPE1, RKI1, TAL1, and TKL1* were constructed for the integration. Subsequently, adaptive evolution was conducted in xylose-rich media under both laboratory conditions and industrial conditions. By utilizing a combinatorial strategy, the best-evolved strain, ABX0928-0630, demonstrated a superior ability to utilize xylose and a higher sugar–alcohol conversion (SAC) efficiency compared to the commercial yeast strain ABX-CIP1 and ABX-CIP2.

## Materials and methods

### Strains, mediums, and culture conditions

The industrial commercial yeast strain *S. cerevisiae* CY used in this study was taken from Biotechnology Center, COFCO Nutrition & Health Research Institute. The commercial–industrial strains of ABX-CIP1 and ABX-CIP2 were introduced by Purdue University and Novozymes Biotechnology Co. Ltd, respectively.

Yeast strains were cultured with either YEPD medium (10 g/l yeast extract, 20 g/l peptone, and 20 g/l glucose), YEPX medium (10 g/l yeast extract, 20 g/l peptone, and 40 g/l xylose), or YNBX medium (0.67% yeast nitrogen base without amino acids and 40 g/l xylose) according to different requirements. Evaluation of flask fermentation was conducted on YEPDX medium (10 g/l yeast extract, 20 g/l peptone, 40 g/l xylose, and 80 g/l glucose). The enzymatic hydrolysis (EH) liquid in the processing of adaptive revolution, and the straw EH liquid extrudate used in the bench- and pilot-scale fermentation were from COFCO Bio-energy (Zhaodong) Co., Ltd. The straw gas explosion material, enzyme preparation and water were mixed into the beaker and enzymatically hydrolyzed in a water bath at 50°C and pH 5.0 for 72 hours to harvest the straw EH solution. NEB® 5-alpha Competent E. coli (New England BioLabs Ltd., Ipswich, MA, USA) was used for plasmid cloning in LB medium (10 g/l NaCl, 5 g/l yeast extract, and 10 g/l tryptone).

### Plasmid and strain construction

XI encoding gene xylA was derived from the *Piromyces* sp. Strain (Lee et al. 2012). The plasmids pUC18-FBA1p/TEF2p-2X-NAT (which contains individual expression cassettes for *xylA* and *XKS1* gene) and pRS42K-TTRR-KanMX (which contains four nonoxidative PPP genes) were constructed by conventional cloning methods. For the pUC18-FBA1p/TEF2p-2X-NAT plasmids, either TEF2p (promoter of *TEF2*) or FBA1p (promoter of *FBA1*) was used to drive *xylA* expression and PGK1p (promoter of *PGK1*) was used to drive *XKS1* expression. Genetic maps of these two plasmids are shown in Figure S1 (Supporting Information).

All strains were constructed or domesticated from the industrial strain *S. cerevisiae* CY. The process of strain construction is depicted in Fig. 1. Given that FPS1 integration site is a single copy number integration site, which affects glycerol efflux protein, inactivating the FPS1 should inhibit glycerol efflux and adjust the direction of carbon metabolism to pyruvate. The repeats of the δ-sites in the genome help with multiple integrations of expression cassettes (Cho et al. 1999, Yamada et al. 2010), the plasmids pRS42K-TTRR-KanMX and pUC18-FBA1p/TEF2p-2X-NAT were orderly recombined into homologous FPS site and δ-site of the *S. cerevisiae* CY genome to avoid plasmid burden.

### Adaptive evolution of engineered strains

For the first 96 generations of adaptive evolution, single colonies were taken from the YEPD solid plate and inoculated into a YEPX (100 ml/250 ml) shake flask containing 40 g/l xylose. After 24 hours of shaking at 30°C, 200 rpm, 1 ml of culture medium was inoculated into fresh YEPX medium. For the later 96 generations of adaptive evolution, 1 ml of culture medium was taken from the former YEPX medium and inoculated into a shaker bottle containing 40 g/l xylose straw gas explosion material (40 ml/100 ml), After 48 hours of shaking at 30°C, 200 rpm, 1 ml of culture medium was taken to inoculate fresh straw gas explosion material. Conditions of strain domestication at various stages are listed in Table S1 (Supporting Information). After every 1–2 months of acclimatization, the fermented liquid is diluted and coated on YNBX plates to isolate the better-performing strain. At each turn, 48 strains were taken to conduct fermentation evaluation to select strains with high XU efficiency and high SAC efficiency for subsequent domestication.

### Flask fermentation evaluation in different stages

Evaluation of early-stage domesticated strains was conducted in synthetic culture medium YEPDX in shake flasks. Yeast seeds were cultured in YEPD at 30°C for 16 hours and then added into shake flasks with an inoculum OD_600nm_ = 1 (0.45 g stem cells/l of fermentation liquor) in 500 ml shake flasks containing 200 ml YEPDX medium. Then cultivated under anaerobic conditions at 30°C for 72 hours. Evaluation of later-stage domesticated strains was conducted in industrial media. Yeast seeds were cultured in YEPX at 30°C for 16 hours and then added into shake flasks with an inoculum OD_600nm_ = 1 in 500 ml shake flasks containing 200 ml straw EH liquid extrudate, 0.5 g/l nitrogen, 50 mg/l penicillin, and 10 mg/l streptomycin. Then cultivated under anaerobic conditions at 30°C for 72 hours. Three parallel essays were set for each group. Glucose, xylose, and ethanol concentrations were detected by HPLC. Titer for ethanol, glucose, xylose, and xylitol were expressed in g/l, yields were expressed in g/g sugar, rates were expressed in g/l/h. The XU and SAC efficiency were calculated according to the following formulas:


\begin{eqnarray*}
{\mathrm{XU\ efficiency}} = {\mathrm{\ }}\frac{{\textit{Initial}\ \textit{Xylose}\ \textit{Content} - End\ \textit{Xylose}\ \textit{Content}}}{{\textit{Initial}\ \textit{Xylose}\ \textit{Content}}} \times 100\%,
\end{eqnarray*}

\begin{eqnarray*}
&&{\mathrm{SAC\ efficiency}}\\
&&\quad = \frac{{End\ \textit{Ethanol}\ \textit{Content} - \textit{Initial}\ \textit{Ethanol}\ \textit{Content}}}{{0.511 \times \left( {\textit{Initial}\ \textit{Glucose}\ and\ \textit{Xylose}\ \textit{Content} - End\ \textit{Glucose}\ and\ \textit{Xylose}\ \textit{Content}} \right)}}\\
&&\quad \times 100\%.
\end{eqnarray*}

XU efficiency represents the consumption of total xylose from the initial xylose concentration. SAC efficiency represents the ratio of ethanol production with sugar consumption according to stoichiometry.

### Pilot-scale fermentation

Pilot-scale fermentation was carried out using 30 m^3^ fermenters. Corn stalks were cut into small sections for soaking in a 20-m^3^ enzymolysis device for 5 minutes, maintaining a pressure of 1.25 M Pa at 190°C for 3 minutes to obtain a corn stover steam explosion material; adjust the moisture content of the steam explosion material to 53wt%, then add 72wt% sulfuric acid solution to mix evenly, add 8wt% of cellulase to EH at 50°C and pH 5.0 for 72 hours to obtain an enzymatically hydrolyzed mature mash. The inoculum was controlled at 0.45 g stem cells/l of fermentation liquor to carry out ethanol fermentation of the enzymatically hydrolyzed mature mash with a loading volume of about 16 m^3^. Fermentation parameters were optimized previously: at the temperature of 32°C, with no aeration, at pH of about 4.5, and penicillin dosage of 90 g to conduct fermentation for 56 hours. Samples were taken every 8 hours to determine the concentration of glucose, xylose, and ethanol concentrations by HPLC. Three parallel assays were set for each fermentation.

### Transcriptome sequencing and data analysis

After incubating in straw EH liquid extrudate at 30°C, samples for transcriptome sequencing were taken at 36 hours. The samples were stored at −80°C until processing. The fragments were sequenced on the BGISEQ-500 platform. There were 21.30 M output data with a comparison rate of 93.02% of each sample generated. HISAT(v2.1.0) was used to map the raw reads to yeast genome. The S288C genome (https://www.ncbi.nlm.nih.gov/genome/?term=s288c) was used as the reference. The ClusterGVis (https://github.com/junjunlab/ClusterGVis) (Li et al. 2023, Zhang 2022) and ClusterProfiler package (Yu et al. 2012) were used for cluster analysis and enrichment analysis to illustrate the trend of relative expression at various domestication stages.

## Results

### Rational construction of xylose fermentation *S. cerevisiae*

Due to the lack of efficient XI, those heterologous isomerases derived from fungi and thermophilic bacteria with high efficiency are applied for constructing xylose metabolic pathway in *S. cerevisiae* (Ha et al. 2011). Here, a 439-amino acid long XI origin from *Piromyces* sp. was selected for modification (Table S2, Supporting Information: SEQ3, SEQ4). A number of potential loci were selected for mutation tests based on the elucidated catalytic mechanism in our previous work (Yi et al. 2019). The mutant with an Ala144Thr amino acid substitution was selected and synthesized to construct the XI expression cassette (Table S2, Supporting Information: SEQ1, SEQ2).

Subsequently, the recombinant plasmid containing the modified *Pi-xylA* gene, *XKS1* and four nonoxidase genes encoding the PPP (*RKI1, RPE1, TKL1*, and *TAL1*) were introduced into the *S. cerevisiae* CY for overexpression (Fig. 1A). The modified strain was cultured and screened in the medium YEPX. Through screening, six recombinant yeast strains (δ-TEF2p-2X-NAT-3#, -4#, -5#, δ-FBA1p-2X-NAT-2#, 3#, and 14#) with the potential to co ferment C5 and C6 sugar to produce ethanol were obtained. The growth curve and XU curve of these six strains were studied, and it was found that these engineering strains had poor adaptability to the environment (Figure S2, Supporting Information). In order to solve this problem, we further domesticated these six strains in YEPDX medium. After seven rounds of domestication, the OD value of δ-TEF2p-2X-NAT-5# strain increased significantly and began to produce odor of alcohol. Therefore, we selected the seventh generation of δ-TEF2p-2X-NAT-5# as the starting strain for the next step of adaptive evolution and renamed it ABX0421 (Fig. 1B).

### Adapted to evolution for rapid xylose fermentation

After seven generations of domestication, the growth rate of δ-TEF2p-2X-NAT-5# strain has been greatly improved (ABX0421), but the growth rate and the XU efficiency are still slow even under aerobic conditions. In order to isolate random mutants with increased growth rate and XU efficiency, ABX0421 strain was continuously transferred in xylose-supplemented YEPX medium under aerobic conditions. After 10 times of transformation, the SAC efficiency of the strain improved from 0% to 37%, and the XU efficiency was improved from 2% to 77% in YEPX medium. But there were no significant trend changes in SAC rate and XU rate in the YEPDX medium, the ethanol yield reached 0.224 g/g sugar with a large amount of xylitol produced, the resultant strain was named ABX0601 (Figure S3, Supporting Information).

**Figure 2.**
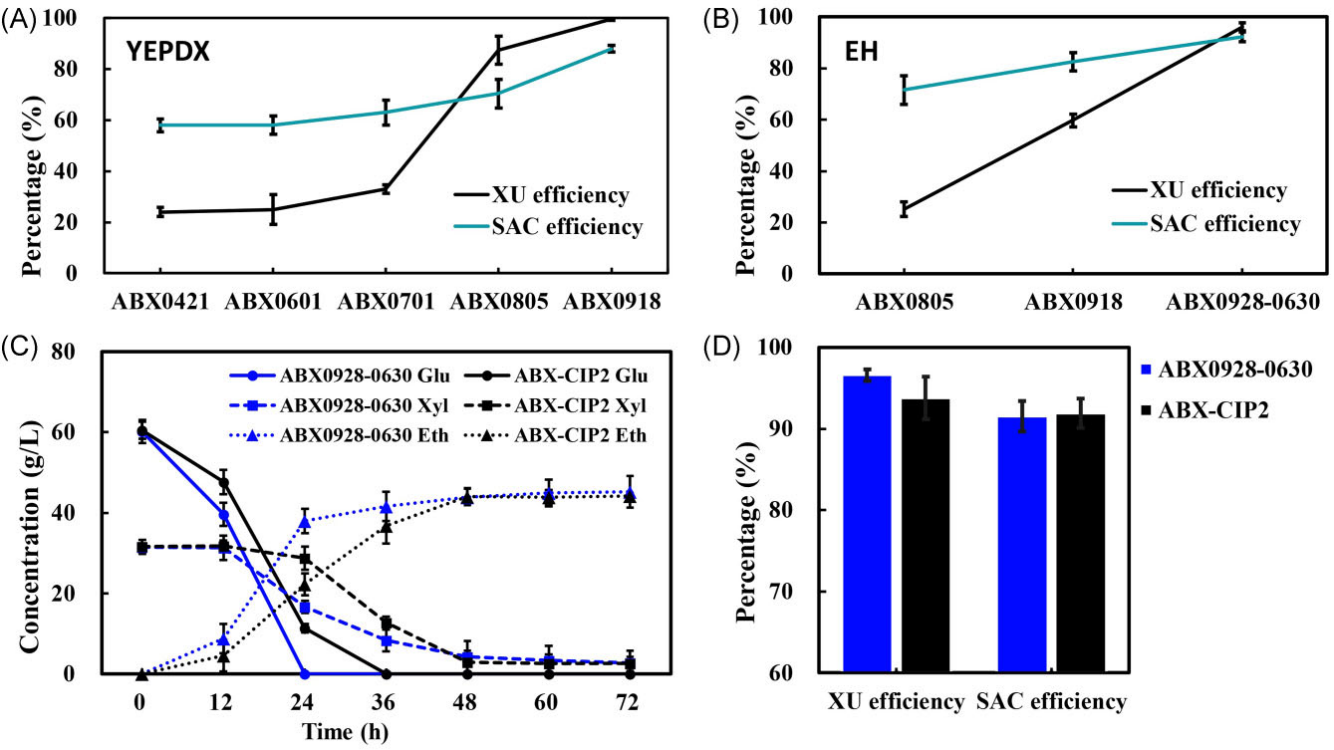
Domestication, screening, and fermentation result of engineered strains. (A) XU and SAC efficiency of strains at different stages in YEPDX medium and (B) EH medium. (C) Glucose, xylose, ethanol concentrations, and (D) XU efficiencies, SAC efficiencies of ABX0928-0630 compared with ABX-CIP2 in EH medium.

Then ABX0601 was further domesticated in YEPX medium for 89 generations and the xylitol production decreased significantly (Fig. 1B; Figure S3, Supporting Information). We took several samples from different stages, including the 17th (ABX0601), 50th (ABX0701), 78th (ABX0805) generations, and 96th (ABX0918). These strains were then subjected to shake flask fermentation experiments in YEPDX medium. Yeast cells were withdrawn at 76 hours for HPLC detection to obtain the fermentation curve and calculate the SAC efficiency and XU efficiency. The results showed that after long-term domestication, the ethanol yield increased from 0.224 g/g sugar to 0.416 g/g sugar, the SAC efficiency increased from 58% to 88%, and the XU efficiency increased from 25% to 99.71% (Fig. 2A; Table S3, Supporting Information). Even though the strain showed excellent fermentation performance in the YEPDX medium, the performance in the EH medium was still not outstanding enough. Hence, to further enhance the performance in EH medium, domestication in industrial conditions was performed by gradually increasing the medium pressure; strains in different stages were taken for the monitoring of characteristics, and the fermentation liquid was diluted and coated on YNBX plates to isolate the best-performing strain (Figure S4, Supporting Information). The strain ABX0928-0630 showed the best performance and the ethanol yield increased from 0.416 g/g sugar to 0.465 g/g sugar, the SAC efficiency increased from 71.56% to 92.31%, the XU efficiency increased from 25.11% to 96.15% in EH medium (Fig. 2B; Table S3, Supporting Information).

To test the superiority of the final domestication strain ABX0928-0630 over a commercialized strain, we performed shake-flask fermentation with the commercial strain ABX-CIP2 as a control in EH medium. The glucose utilization efficiency and the XU efficiency of ABX0928-0630 were higher than those of ABX-CIP2, while the SAC efficiency of ABX0928-0630 was slightly inferior (Fig. 2C and D), indicating a preferential performance of the domesticated strain over ABX-CIP2. In addition, ABX0928-0630 took less time to reach a stable value during fermentation, which can effectively save time in commercial industrial cellulose ethanol fermentation.

Besides, based on the performance of strains reported in the last 10 years, the ethanol productivity and XU achieved in this research demonstrate a clear advantage compared to most of the studies reported in Table 1. When cultured in the YEPDX medium, the engineered strain shows a high xylose consumption rate and ethanol productivity in 24 hours compared with other studies. Although the ethanol productivity of this strain with straw EH liquid extrudate during the first 24 hours is low, which is caused by the stresses from the medium, the engineered strain still demonstrates a rewarding performance of ethanol yield as well as XU and SAC efficiency. It's worth mentioning that the xylitol accumulation of the engineered strain in this research is very low no matter in YEPDX medium or industrial straw EH liquid extrudate medium, which helps avoid production inhibition by xylitol.

**Table 1.** Main fermentation performance indexes for the present study and reported studies

Sugar (g/l)	Y_EtOH_ (g/g sugar)	Y_xylitol_ (g/g sugar)	24h-C_xylose_ (g/l/h)	24h-P_EthOH_ (g/l/h)	Medium	Reference
71.93G + 39.87X	0.415	✘	1.512	1.679	YEPDX	This research
78.6G + 30.3X	0.465	✘	0.292	1.783	Straw EH liquid extrudate	This research
60X	0.31	✔	1.75	0.69	YEPX	Kobayashi et al. (2017)
85G + 35X	0.46	✘	0.94	N.A.	YEPDX	Kobayashi et al. (2018)
50X	0.35	0.11	N.A.	N.A.	YEPX	Osiro et al. (2019)
70G + 40X	0.41	✔	N.A.	0.85	YEPDX	Kim et al. (2013b)
20G + 50X	0.25	✔	0.842	0.208	Pitch pine hydrolysates	Jo et al. (2016)
40X	0.32	0.01	N.A.	N.A.	YEPX	Jeong et al. (2020)
65X	0.38	N.A.	N.A.	0.87	Chemically defined medium	Sandri et al. (2023)
20G + 80X	0.27	0.18	1.04	0.62	YEPDX	Thalita Peixoto et al. (2022)
120X	0.33	✔	N.A.	N.A.	Corn hydrolysates	Li et al. (2022)
50G + 50X	0.29	0.03	N.A.	N.A.	YEPDX	Liu et al. (2010)

Sugar: G for glucose and X for xylose; XU: xylose utilization; Y: yield, g/g sugar; P: productivity, g/l/h; and C: consumption rate, g/l/h. For Y_xylitol_, all yields less than 0.01 were marked as ✘ and higher than 0.01 were marked as ✔.

### Pilot-scale fermentation compared with commercial strains

To evaluate the fermentation performance of ABX0928-0630 in a larger industrial fermentation environment, we conducted a fermentation test of ABX0928-0630 with real material in a pilot-scale fermenter (30 m³) at a biochemical factory (COFCO, Zhao Dong) and compared with two commercial industrial production strains (ABX-CIP1 and ABX-CIP2). Samples were taken every 8 hours to determine the sugar alcohol concentration of the mash (Figure S5 and Table S4, Supporting Information).

**Figure 3.**
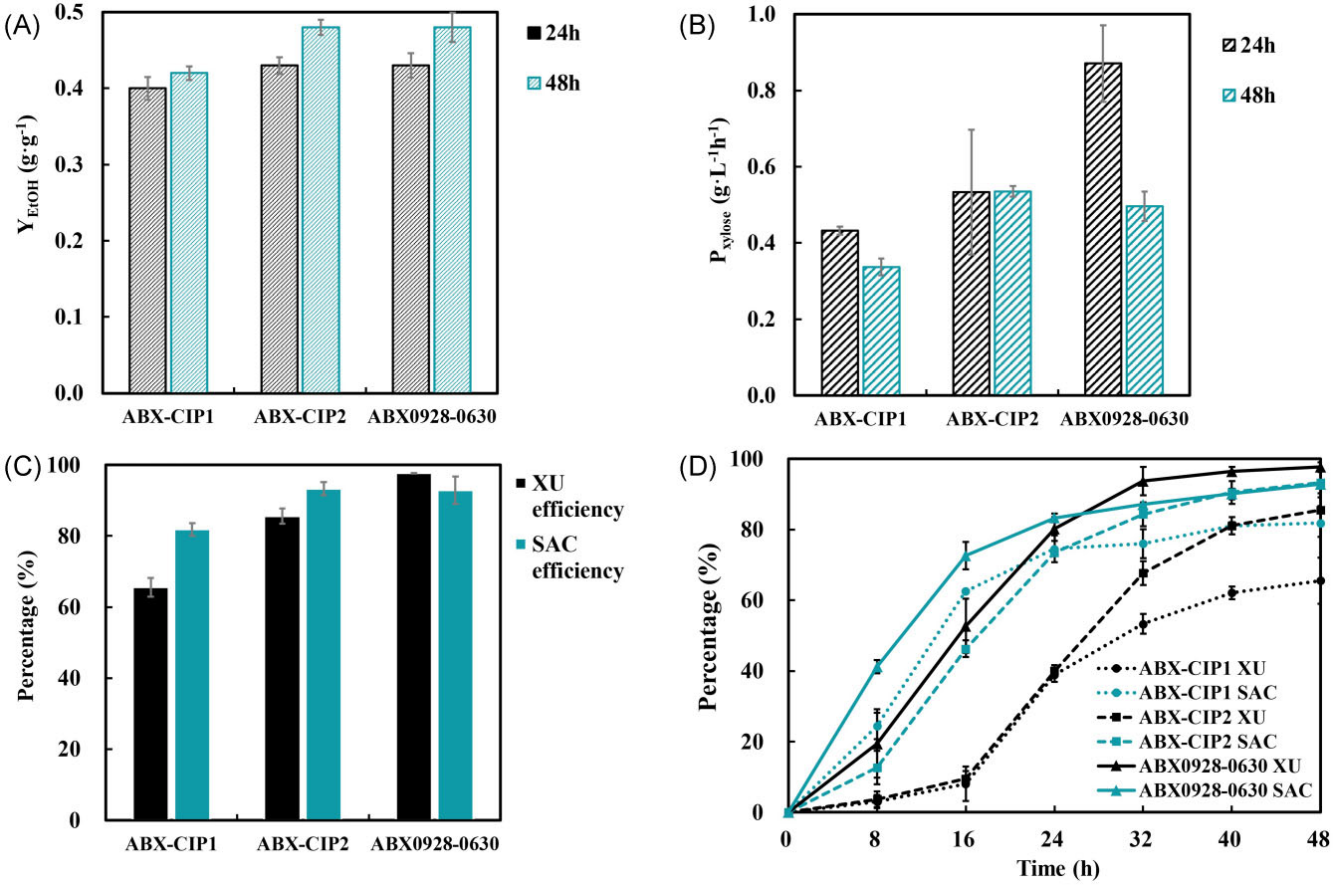
Pilot-scale fermentation of ABX0928-0630 compared with commercial strains ABX-CIP1 and ABX-CIP2. (A) Bar chart of the ethanol yield within 24 and 48 hours and (B) xylose consumption rate within 24 and 48 hours. (C) Bar chart of the XU efficiency and SAC efficiency. (D) The time–rate curve of the total SAC efficiency and XU efficiency.

The fermentation data of the three strains showed that the final strain ABX0928-0630 after adaptive evolution can convert 56.43 g/l glucose and 24.35 g/l xylose to 38.25 g/l ethanol in 48 hours at an initial OD600 of 1.0 (0.63 g DCW/l) without producing xylitol, the ethanol yield reached up to 0.48 g/g sugar, which is higher or the same level as the two commercial strains ABX-CIP1 and ABX-CIP2 (0.42 g/g sugar and 0.48 g/g sugar, respectively) (Fig. 3A). The volumetric xylose consumption of ABX0928-0630 during the first 24 hours could reach up to 0.76 g/l/h, which is higher than that of ABX-CIP1 and ABX-CIP2 (0.43 g/l/h and 0.53 g/l/h, respectively) (Fig. 3B). The average XU efficiency of ABX0928-0630 reached 97.7%, which is much higher than that of ABX-CIP1 (65.6%) and ABX-CIP2 (85.6%). The average SAC efficiency of ABX0928-0630 was 92.9%, while that of the ABX-CIP1 and ABX-CIP2 were 81.9% and 93.3% respectively (Fig. 3C). The time–rate curves of both the total SAC efficiency and XU efficiency shown in Fig. 3(D) show the same trend: ABX0928-0630 has the highest total SAC and XU efficiency, followed by ABX-CIP2, and the lowest was exhibited by ABX-CIP1. The fermentation with ABX0928-0630 in the pilot-scale test was better than fermentations with other strains, indicating that this strain is suitable for pilot production and has production capacity for industrial applications.

### Transcriptome analysis throughout domestication

Through metabolic engineering, we enhanced the adaptation of *S. cerevisiae* growth on xylose with glucose. However, it was still not clear how the cellular metabolism was reshaped for this change. To explore the gene expression in the cellulose metabolism pathway at different stages of adaptive evolution, strains at different stages of domestication (represent the initial industrial strain, the initial domesticating strain, the final strain domesticated in synthetic medium and the final strain domesticated in industrial medium, respectively) were selected for transcriptome sequencing. Based on previously obtained fermentation data, the yeast strains can run out of glucose and begin to utilize xylose between 24 and 48 hours. We presume that this stage may be when cellulose metabolism is most active and the relevant gene expression in the pathway is the highest. Thus, we performed shake-flask fermentation in EH medium with these strains and withdrew the cells at 36 hours for RNA sequencing.

**Figure 4.**
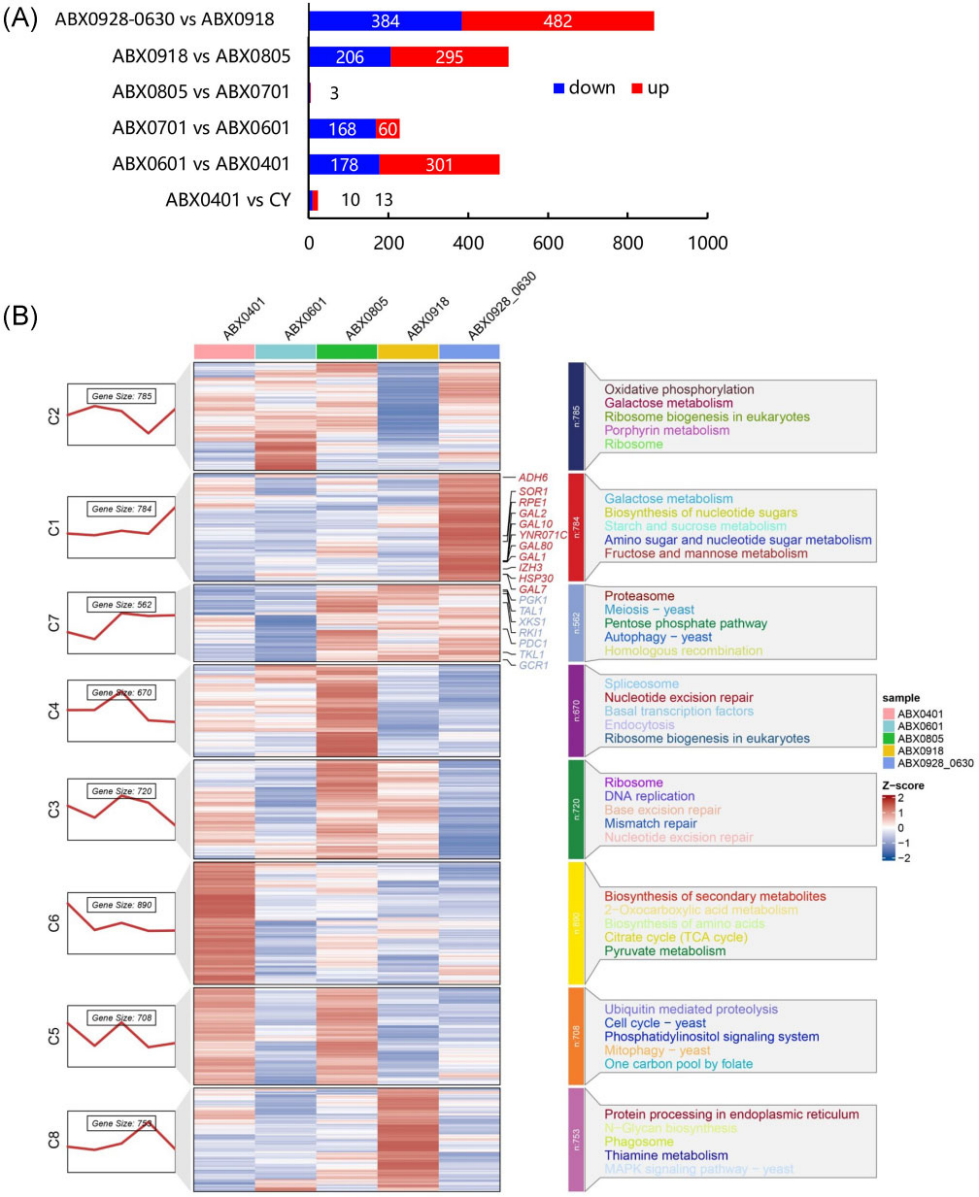
(A) The number of differentially expressed genes (DEGs) (bcv = 0.2, *P* < .05) during different stages of domestication. (B) Differential expression patterns across different domestication stages. The DEGs between ABX0401, ABX0601, ABX0805, ABX0918, and ABX0928-0630 were classified into eight clusters, C1–C8 representing different patterns in response to domestication. Gene size indicates the number of genes in each pattern. The genes and pathways highlighted with different colors in each cluster possessed distinct functions which were revealed by KEGG pathway enrichment analysis.

To comprehensively understand the entire process of adaptive evolution and reveal previously uncovered traits related to xylose metabolism, we used all transcripts to perform principal component analysis. As shown in Fig. 4(A), good agreement between ABX0401 and CY, ABX0805 and ABX0701 suggesting the transcriptional similarity of physiological status in each pair. To define the temporal characteristics of the complete transcriptome dataset, we performed clustering analysis across ABX0401, ABX0601, ABX0805, ABX0918, and ABX0928-0630. Among the total expressed genes, eight clusters exhibited distinct patterns among the five groups were identified (Fig. 4B). The genes in each cluster possessed distinct functions as revealed by KEGG pathway enrichment analysis.

We first analyzed the regulation of relevant genes in several metabolic pathways that are most pertinent to xylose metabolism (Table S5, Supporting Information). Genes in clusters C1 and C7 showed a significant increase during domestication, and the functions of these genes were enriched in galactose metabolism, biosynthesis of nucleotide sugars, starch and sucrose metabolism, amino sugar and nucleotide sugar metabolism, fructose and mannose metabolism, proteasome, and meiosis. The externally integrated gene *XKS1*, responsible for xylulose phosphorylation, showed a significant increase in transcription levels through domestication. Continuing with the conversion of xylulose-5P into the PPP, the externally integrated TTRR (*TKL1, TAL1, RPE1*, and *RKI1*) genes also showed a substantial improvement in transcription. Subsequently, within the metabolic pathway from Glyceroldehyde-3P to ethanol, most genes exhibited upregulation, such as *PGK1, PYK1*, and *PDC1* and *ADH6* (Fig. 4B). As mentioned above, xylose transportation relies on hexose transporters, genes related to this, including *SOR1* and *YNR071C* showed a significant increase which suggests an increased efficiency of sugar uptake during domestication. *IZH3, GCR1*, and *HSP30*, which are related to sugar sensing and signalomics were upregulated, indicating an enhanced ability of the engineered strains for sugar sensing and ethanol response.

Notably, genes in cluster C6 were enriched in the biosynthesis of secondary metabolites, including 2-oxocarboxylic acid metabolism, biosynthesis of amino acids, TCA cycle and pyruvate metabolism, which aligns with our previous findings. This implies that the increased utilization of xylose by yeast had an impact on cellular activity, ultimately leading to a reduction in secondary metabolism. In pathways leading to secondary metabolites, most of the TCA cycle genes show significant downregulation. This downregulation prevents the accumulation of acetate byproducts and helps maintain pH levels. While the genes associated with glycerolipid metabolism generally exhibit an upregulation trend, although many of them are subsequently downregulated as domestication progresses. We propose that these trends are a result of osmotic regulation and energy optimization within the cell. During the adaptive domestication, the composition of the culture medium changed from a mixture of glucose and xylose to xylose alone and then to industrial materials, transitioning from relatively mild to complex and severe conditions. Consequently, the initial increase in glycerol production could be viewed as a protective response to the more severe living conditions. As the domestication process continued, the engineered strain gradually adapted to the harsher growth environment and tended to adopt a more energy-efficient production mode to achieve balance, resulting in the downregulation of these genes. The optimization ability is mainly due to adaptive domestication in industrial conditions and not only maintains a transcription balance but also avoids metabolic disturbances with multiple carbon resources (Infante et al. 2003).

Another major problem in simultaneous cofermentation of glucose and xylose is the inhibition effect of carbon decomposition (CCR effect). Xylose transportation relies on hexose transporters, however, xylose fermentation is not as efficient as glucose fermentation in *S. cerevisiae*. The significantly increased transcript levels of a series of *GAL* genes in galactose metabolism pathway, such as *GAL1, GAL2, GAL7, GAL10*, and *GAL80*, are involved in glucose-repressible galactose metabolism. Their gratuitous induction suggests a probable loss of glucose repression (Baleja et al. 1997).

## Discussion

Xylose is the second-highest monosaccharide in lignocellulose hydrolysate. In the production of cellulose ethanol using lignocellulose hydrolysate as raw material, efficient and full utilization of xylose is an essential characteristic of fermentation strains. However, natural brewing yeast cannot metabolize xylose. Therefore, obtaining yeast strains that can efficiently metabolize xylose through engineering modification of brewing yeast is a key issue in cellulose ethanol production. Previous studies have been focused on hosts of laboratory haploid strains and expression based on plasmids, which were ineffective in actual industrial production (Selim et al. 2018). In this study, the industrial diploid strain was chosen as the chassis host, and genes related to XU were integrated into yeast chromosomes. In the following, adaptive evolution, real industrial fermentation and transcriptome analysis were conducted sequentially. Our results indicate that the best-evolved strain demonstrated a superior ability to utilize xylose and a higher SAC efficiency compared to the commercial yeast strain.

Transcriptome data showed a significant increase in the expression level of hexose transporters, indicating an increased efficiency in sugar uptake during domestication. The genes related to sugar sensing and signal omics were upregulated, indicating an enhanced ability of the engineered strain to respond to sugar sensing and ethanol. The expression levels of other genes related to processes such as galactose metabolism, nucleotide sugar biosynthesis, starch and sucrose metabolism, amino and nucleotide sugar metabolism, fructose and mannose metabolism, proteasome, and meiosis also significantly increased during domestication. The increase in XU by yeast has an impact on cell activity, ultimately leading to a decrease in secondary metabolism. This downregulation can prevent the accumulation of acetate byproducts and help maintain pH levels. With the progress of domestication, genes related to glycerol lipid metabolism show a trend of increasing first and then decreasing, which may be related to intracellular osmotic regulation and energy optimization. We propose that these trends are a result of osmotic regulation and energy optimization within the cell. During the adaptive domestication, the composition of the culture medium changed from a mixture of glucose and xylose to xylose alone and then to industrial materials, transitioning from relatively mild to complex and severe conditions. Consequently, the initial increase in glycerol production could be viewed as a protective response to the more severe living conditions. As the domestication process continued, the engineered strain gradually adapted to the harsher growth environment and tended to adopt a more energy-efficient production mode to achieve balance, resulting in the downregulation of these genes. The optimization ability is mainly due to adaptive domestication in industrial conditions and not only maintains a transcription balance but also avoids metabolic disturbances with multiple carbon resources (Infante et al. 2003). Galactose transporters can transport xylose without being inhibited by glucose, and xylose transporters can achieve simultaneous consumption of glucose and xylose, which provides the possibility of achieving efficient coutilization of glucose and xylose.

## Conclusions

Through genetic modification and continuous domestication, we have obtained the engineered strain ABX0928-0630, and the fermentation of corn stalk-based raw materials with ABX0928-0630 increased the ethanol yield by ~30% in 30 m^3^ fermenters under pilot-scale conditions, decreasing the cost of raw material by about $150–175 per ton ethanol. With a shorter fermentation time and higher ethanol conversion efficiency and XU efficiency, the strain ABX0928-0630 can effectively decrease production cost and time, which indicates a better commercial application prospect in ethanol fermentation.

Compared with the XR-XDH pathway, the XI pathway has a higher ethanol yield during xylose fermentation. In this study, we constructed a recombinant strain, but further research is needed on the integration sites of genes on the genome and how to improve enzyme activity. Moreover, this study explains the changes in gene expression during the process of modification and domestication at the transcriptome level, and these research findings can be used to guide future genetic modification work. In addition, glucose and xylose are the two main components of lignocellulose. The simultaneous consumption of glucose and xylose is crucial for the production of fuel and chemicals using lignocellulose hydrolysis products as raw materials. In future research, further attention needs to be paid to the issue of gene transcription inhibition related to xylose metabolism, as well as the exploration of new xylose transporters, in order to achieve efficient coutilization of glucose and xylose.

## Supplementary Material

foae001_Supplemental_Files
