# Supplementary material for: Construction of an economical xylose-utilizing Saccharomyces cerevisiae and its ethanol fermentation
Source: FEMS Yeast Res. 2024 Jan 24;24:foae001. doi: 10.1093/femsyr/foae001 (PMC10855017; doi:10.1093/femsyr/foae001)
Supplement: foae001_Supplemental_Files [file foae001_supplemental_files.zip › Supplimental files-v2.docx]

**Supporting Information**

**Construction of an Economical Xylose-utilizing *Saccharomyces cerevisiae* and Its Ethanol Fermentation**

Fan Li^a,b,c,#^, Wenxin Bai^a,#^, Yuan Zhang^a,c^, Zijian Zhang^a^, Deguo Zhang^c,d^, Naidong Shen^a,c^, Jingwei Yuan^b,c^, Guomiao Zhao^a,c,*^, Xiaoyan Wang^a,c,*^

*^#^ Equal contribution*

*^a^ Nutrition & Health Research Institute, COFCO Corporation, Beijing 102209, China*

*^b^  COFCO Biochemical and Bioenergy (Zhaodong) Co., Ld., Suihua 151100, China*

*^c^ COFCO Corporation, Beijing 100020, China*

*^d^ COFCO Biotechnology Co., Ltd., Bengbu 233010, Anhui, China*


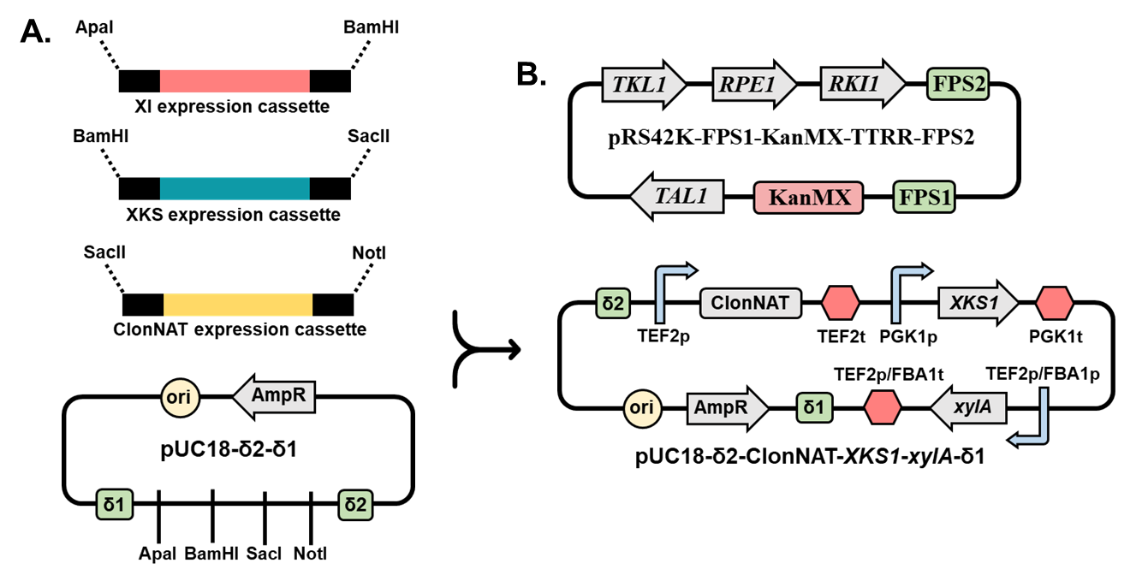


Figure S1 Gene map of plasmids pUC18-FBA1p/TEF2p-2X-NAT and pRS42K-TTRR-KanMX for improving the yeast strain *S.cerevisiae* CY. (A) XI, XKS, and antibiotic ClonNAT expression cassettes are integrated into the plasmid pUC18 flanked with delta sites to construct the recombinant plasmid pUC18-FBA1p/TEF2p-2X-NAT (Puc18-δ2-ClonNAT-XKS1-xylA-δ1). (B) The same strategy is applied for the construction of the plasmid pRS42K-TTRR-KanMX (pRS42K-FPS1-KanMX-TTRR-FPS2). For the pUC18-FBA1p/TEF2p-2X-NAT plasmids, either TEF2p (promoter of *TEF2*) or FBA1p (promoter of *FBA1*) was used to drive *xylA* expression and PGK1p (promoter of *PGK1*) was used to drive *XKS1* expression.

**
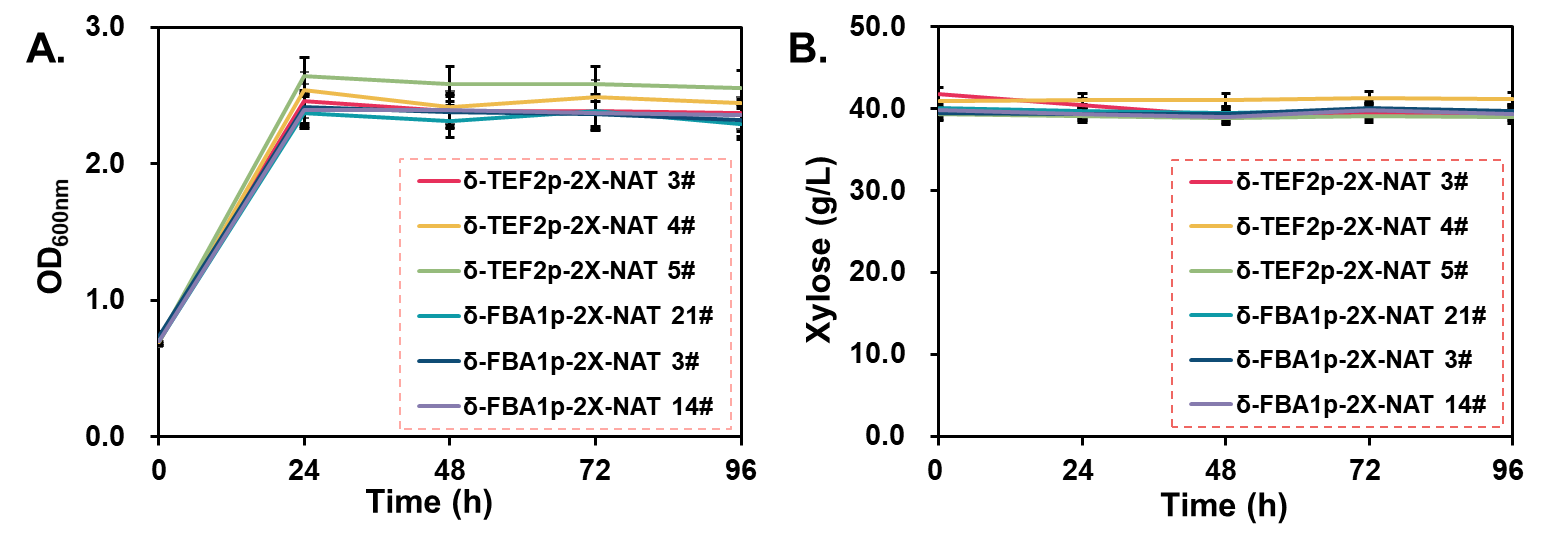
**

Figure S2 Fermentation curve of δ-TEF2p-2X-NAT(3#, 4#, 5#) and δ-FBA1p-2X-NAT(21#, 3#, 14#). (A) Under the condition of low initial inoculation density (about 0.7), the yeast density only reached and maintained at a very low level, indicating that the strain no longer grew after 24 hours. (B) Xylose was seldom utilized no matter which strain in this test, the largest consumption is only 2 g/L of xylose.


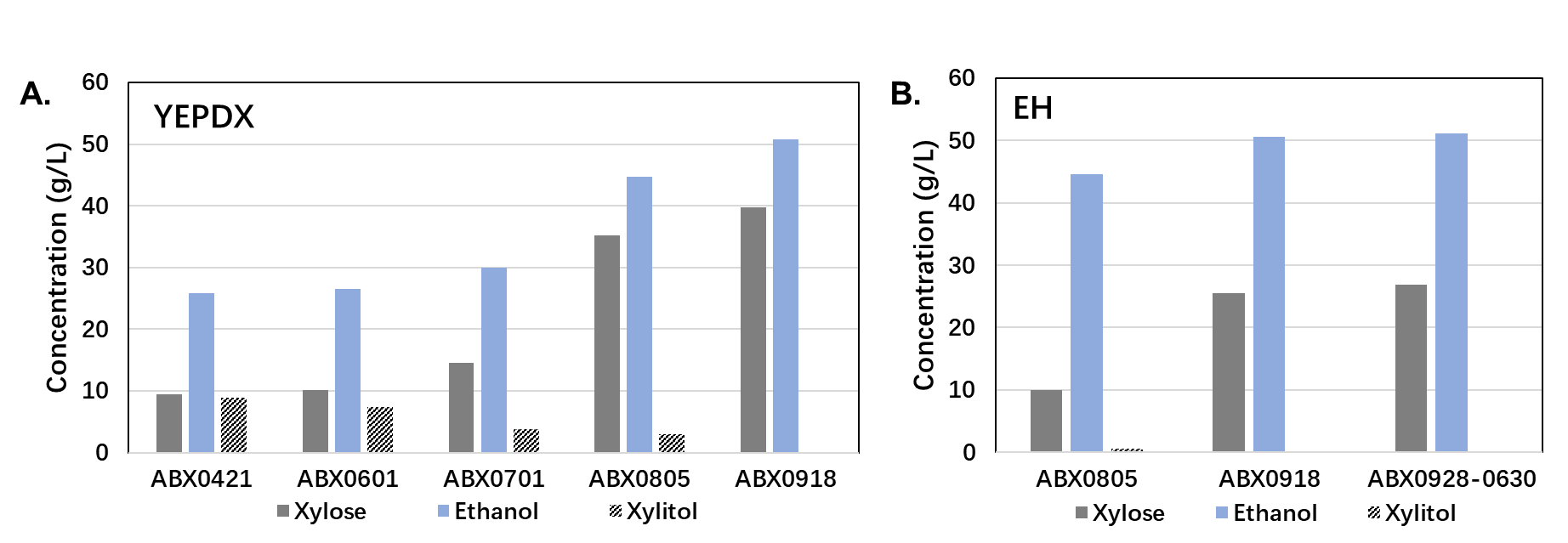


Figure S3 Fermentation results of strains in various stages under (A) synthetic culture medium YEPDX and (B) industrial medium EH domestication. The legend of xylose represents the total consumption of xylose, legends of ethanol and xylitol represent the total production of ethanol and xylitol.


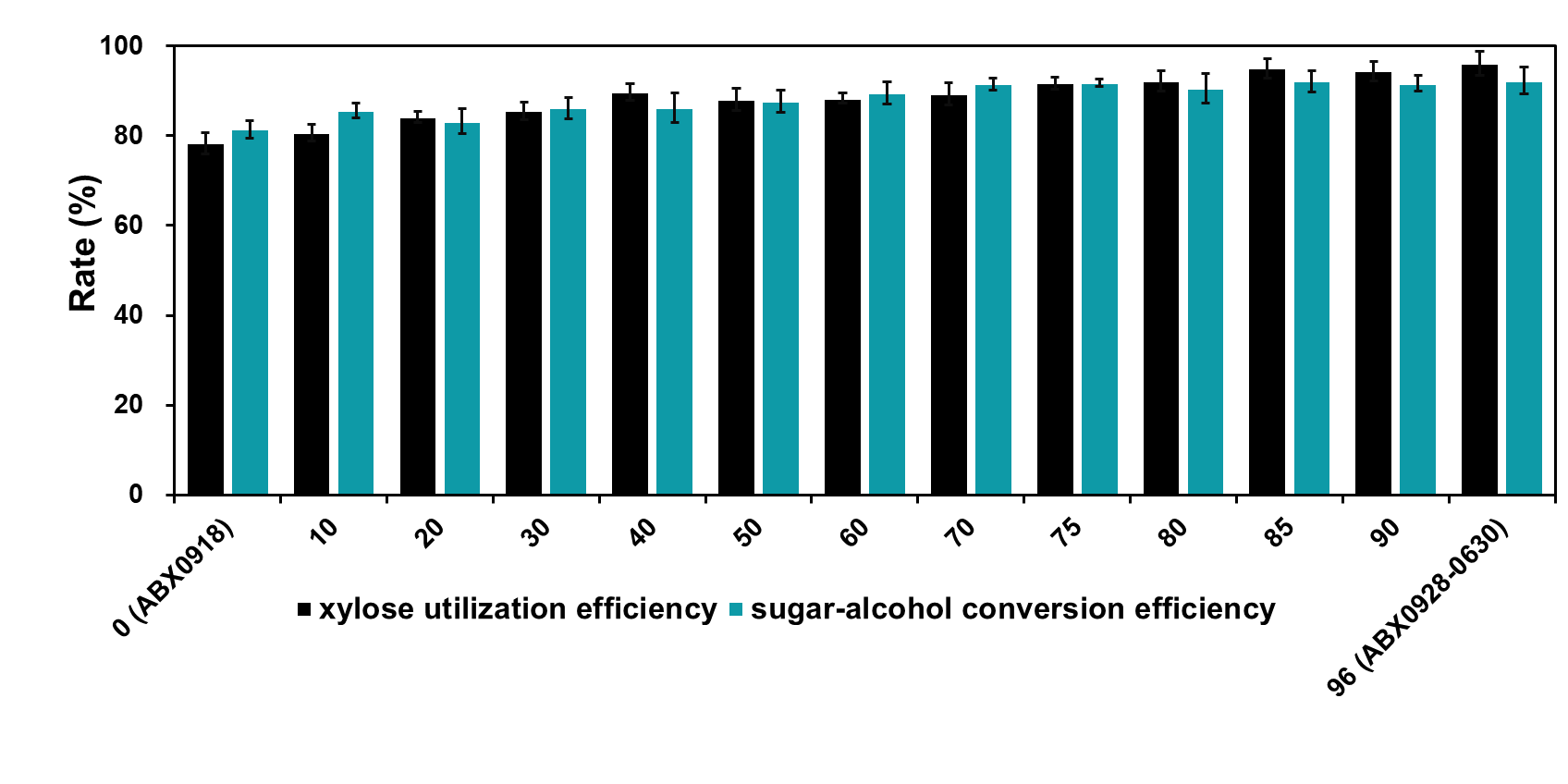


Figure S4 Characteristics of strains in various stages under industrial culture medium domestication. The starting strain was named ABX0918 and the final strain was named ABX0928-0630.


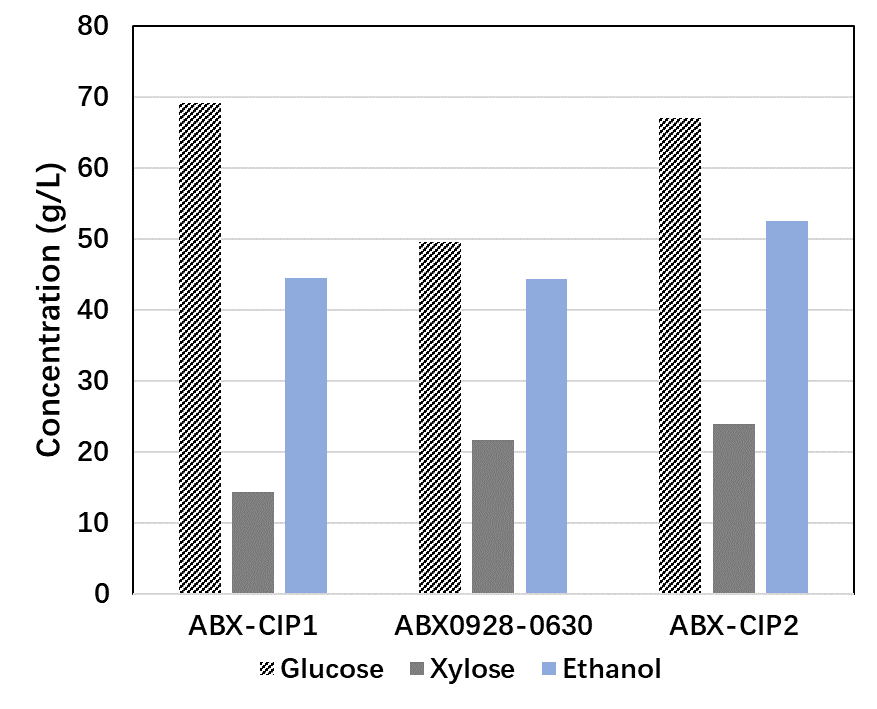


Figure S5 Pilot scale fermentation curve of ABX0928-0630 compared with commercial strains ABX-CIP1 and ABX-CIP2. The legend of glucose and xylose represents the total consumption of glucose and xylose, legend of ethanol represents the total production of ethanol.

Table S1 Conditions of strain domestication at various stages

| First 96 generations of domestication in synthetic medium (From ABX0421 to ABX0918) | | |
| --- | --- | --- |
| 1-7 | 5ml 20g / L xylose YEPX | conducted every three days |
| 8-17 | 5ml 40g/L YEPX | conducted everyday |
| 18-50 | 50ml 40g / L xylose YEPX | gas-permeable membranes |
| 51-78 | 50ml 40g / L xylose YEPX | micro-aerobic condition |
| 79-96 | 50ml 40g / L xylose 0.67% YNBX | micro-aerobic condition |
| Later 96 generations of domestication in EH medium (From ABX0928 to ABX0928-0630) | | |
| 1-3 | 50% EH + 50% YEPDX | 10% inoculum size |
| 7-36 | 75% EH + 25% YEPDX | 10% inoculum size |
| 41-87 | 100% EH | 10% inoculum size |
| 88-96 | 100% EH | 5% inoculum size |

Table S3 Fermentation performance of engineered strain during evolution

| Strain | Medium | Sugar (g/L) | Fermentation length | T_xylose_  (g·L^-1^) | T_ethanol_  (g·L^-1^) | Y_ethanol_  (g·g^-1^) | T_xylitol_  (g·L^-1^) | 24h-P_xylose_  (g·L^-1^h^-1^) | 24h-C_ethanol_  (g·L^-1^h^-1^) | 72h-P_xylose_  (g·L^-1^h^-1^) | 72h-C_ethanol_  (g·L^-1^h^-1^) |
| --- | --- | --- | --- | --- | --- | --- | --- | --- | --- | --- | --- |
| A0601 | YEPX | 40.5X | 96h | 30.87 | 5.87 | 0.145 | 3.91 | 0.061 | 0 | 0.359 | 0.088 |
| A0601 | YEPDX | 78.6G+40.5X | 96h | 10 | 26.65 | 0.224 | 7.31 | 0.130 | 1.406 | 0.139 | 0.41 |
| A0701 | YEPDX | 78.13G+43.49X | 76h | 14.5 | 30.02 | 0.247 | 3.82 | 0.263 | 1.531 | 0.236 | 0.391 |
| A0805 | YEPDX | 82.01G+40.27X | 76h | 35.2 | 44.71 | 0.366 | 3.04 | 0.738 | 1.753 | 0.489 | 0.621 |
| A0805 | EH | 84.24G+39.66X | 76h | 10.46 | 44.61 | 0.36 | 0.07 | 0.048 | 1.616 | 0.145 | 0.62 |
| A0918 | YEPDX | 71.93G+39.87X | 72h | 39.87 | 46.4 | 0.415 | 0 | 1.512 | 1.679 | 0.554 | 0.644 |
| A0918 | EH | 80.43G+39.31X | 72h | 23.34 | 49.85 | 0.416 | 0 | 0.173 | 1.75 | 0.324 | 0.692 |
| A0928-0630 | EH | 78.6G+30.3X | 72h | 19.8 | 50.7 | 0.465 | 0 | 0.292 | 1.783 | 0.275 | 0.704 |
| CIP2 | EH | 78.8G+31.1X | 72h | 25.8 | 51.5 | 0.469 | 0 | 0.038 | 1.312 | 0.358 | 0.715 |

* Txylose, Tethanol, and Txylitol represent the titre of xylose consumption, ethanol production and xylitol production after fermentation. Yethanol represents the yield of ethanol per g of sugar during fermentation. C_xylose_ and P_ethanol_ represent the volumetric consumption rate or productivity of xylose and ethanol during 24 hours / 72 hours culturing. For sugar concentrations, G represents glucose and X represents xylose.

Table S4 Pilot scale fermentation data of ABX0928-0630 compared with commercial strain CIP1 and CIP2

| Strain | Time(h) | T_Glucose_ (g·L^-1^) | T_Xylose_ (g·L^-1^) | C_xylose_  (g·L^-1^h^-1^) | T_EtOH_ (g·L^-1^) | Y_EtOH_  (g·g^-1^) | P_EtOH_  (g·L^-1^h^-1^) | SAC (%) | XU (%) |
| --- | --- | --- | --- | --- | --- | --- | --- | --- | --- |
| CIP1 | 0 | 77.325 | 24.63 | - | 5.45 | - | - |  |  |
|  | 24 | 0 | 14.25 | 0.43 | 40.90 | 0.40 | 1.48 | 75.535 | 42.11865 |
|  | 48 | 0 | 8.45 | 0.34 | 44.45 | 0.42 | 0.81 | 81.8603 | 65.5874 |
| CIP2 | 0 | 73.575 | 30.10 | - | 5.25 | - | - |  |  |
|  | 24 | 0.25 | 17.31 | 0.53 | 42.10 | 0.43 | 1.54 | 76.46 | 44.0668 |
|  | 48 | 0 | 4.42 | 0.54 | 52.45 | 0.48 | 0.98 | 93.302 | 85.59335 |
| ABX0928-0630 | 0 | 56.425 | 24.35 | - | 6.10 | - | - |  |  |
|  | 24 | 0 | 6.15 | 0.87 | 38.85 | 0.43 | 1.36 | 89.68 | 75.9558 |
|  | 48 | 0 | 0.55 | 0.50 | 44.35 | 0.48 | 0.80 | 92.902 | 97.73685 |

*All data were calculated as the average value of replicates.

* T_xylose_ and T_EtOH_ represent the titre of xylose consumption and ethanol production after fermentation. Y_EtOH_ represents the yield of ethanol per g sugar during fermentation. C_xylose_ and P_EtOH_ represent the volumetric consumption rate or productivity of xylose and ethanol during 24 hours / 48 hours of culturing. For sugar concentrations, G represents glucose and X represents xylose.

Table S2 Original and modified gene sequence and amino acid sequence of xylA

| SEQ 1:  Artificial Sequence (Ala144  Thr mutant amino acid sequence) | Met Ala Lys Glu Tyr Phe Pro Phe Thr Gly Lys Ile Pro Phe Glu Gly Lys Glu Ser Lys Asn Val Met Ala Phe His Tyr Tyr Asp Pro Glu Lys Val Val Met Gly Lys Lys Met Lys Asp Trp Leu Lys Phe Ala Met Ala Trp Trp His Thr Leu Gly Gly Ala Ser Ala Asp Gln Phe Gly Gly Gln Thr Arg Ser Tyr Glu Trp Asp Lys Ala Glu Asp Ala Val Gln Arg Ala Lys Asp Lys Met Asp Ala Gly Phe Glu Ile Met Asp Lys Leu Gly Ile Glu Tyr Phe Cys Phe His Asp Val Asp Leu Val Glu Glu Gly Ala Th Ile Ala Glu Tyr Glu Glu Arg Met Lys Ala Ile Thr Asp Tyr Ala Gln Glu Lys Met Lys Gln Phe Pro Asn Ile Lys Leu Leu Trp Gly Thr Thr Asn Val Phe Gly Asn Lys Arg Tyr Ala Asn Gly Ala Ser Thr Asn Pro Asp Phe Asp Val Val Ala Arg Ala Ile Val Gln Ile Lys Asn Ala Ile Asp Ala Thr Ile Lys Leu Gly Gly Thr Asn Tyr Val Phe Trp Gly Gly Arg Glu Gly Tyr Met Ser Leu Leu Asn Thr Asp Gln Lys Arg Glu Lys Glu His Met Ala Thr Met Leu Thr Met Ala Arg Asp Tyr Ala Arg Ala Lys Gly Phe Lys Gly Thr Phe Leu Ile Glu Pro Lys Pro Met Glu Pro Ser Lys His Gln Tyr Asp Val Asp Thr Glu Thr Val Ile Gly Phe Leu Arg Ala His Gly Leu Asp Lys Asp Phe Lys Val Asn Ile Glu Val Asn His Ala Thr Leu Ala Gly His Thr Phe Glu His Glu Leu Ala Cys Ala Val Asp Ala Gly Met Leu Gly Ser Ile Asp Ala Asn Arg Gly Asp Ala Gln Asn Gly Trp Asp Thr Asp Gln Phe Pro Ile Asp Asn Phe Glu Leu Thr Gln Ala Met Leu Glu Ile Ile Arg Asn Gly Gly Leu Gly Asn Gly Gly Thr Asn Phe Asp Ala Lys Ile Arg Arg Asn Ser Thr Asp Leu Glu Asp Leu Phe Ile Ala His Ile Ser Gly Met Asp Ala Met Ala Arg Ala Leu Glu Asn Ala Ala Ala Ile Leu Glu Glu Ser Glu Leu Pro Ala Met Lys Lys Glu Arg Tyr Ala Ser Phe Asp Ser Gly Ile Gly Lys Asp Phe Glu Glu Gly Lys Leu Thr Leu Glu Gln Ala Tyr Glu Tyr Gly Lys Lys Val Glu Glu Pro Lys Gln Ile Ser Gly Lys Gln Glu Lys Tyr Glu Thr Ile Val Ala Leu Tyr Cys Lys |
| --- | --- |
| SEQ 2: Artificial Sequence  (Ala144  Thr mutant gene sequence) | ATGGCAAAAG AGTATTTTCC GTTTACCGGT AAGATTCCTT TCGAAGGAAA AGAGAGCAAG AACGTGATGG CATTCCACTA TTATGACCCC GAGAAGGTTG TGATGGGAAA GAAGATGAAA GACTGGCTGA AGTTTGCCAT GGCATGGTGG CACACCCTGG GTGGCGCTTC TGCAGACCAG TTTGGCGGAC AGACCCGCTC CTATGAGTGG GACAAGGCTG AGGACGCTGT TCAGCGTGCC AAGGACAAGA TGGACGCCGG TTTCGAGATC ATGGACAAGC TCGGCATCGA ATATTTCTGC TTCCACGACG TGGACCTCGT AGAGGAGGGC GCCACCATCG CAGAGTATGA GGAGCGCATG AAGGCCATTA CTGACTATGC TCAGGAGAAG ATGAAGCAGT TCCCCAACAT CAAGTTGCTG TGGGGTACCA CCAATGTCTT TGGTAACAAG CGCTATGCCA ACGGTGCTTC CACCAATCCC GACTTCGACG TAGTGGCACG TGCTATCGTC CAGATCAAGA ACGCTATCGA CGCCACCATC AAGCTCGGCG GCACCAACTA TGTGTTCTGG GGCGGACGTG AGGGCTATAT GAGCCTCCTC AACACCGACC AGAAGCGTGA GAAGGAGCAC ATGGCCACCA TGCTGACCAT GGCCCGCGAC TACGCACGCG CAAAGGGCTT CAAGGGCACC TTCCTCATCG AGCCGAAACC CATGGAGCCT TCCAAGCACC AGTATGATGT CGACACTGAG ACTGTGATCG GATTCCTCCG CGCTCATGGA CTCGACAAGG ACTTCAAGGT GAACATCGAG GTGAACCACG CCACATTGGC CGGCCACACC TTCGAGCACG AACTGGCTTG CGCCGTGGAT GCCGGTATGC TGGGTTCAAT CGACGCCAAC CGTGGTGACG CTCAGAACGG CTGGGATACC GACCAGTTCC CCATCGACAA CTTCGAACTC ACACAGGCTA TGCTGGAGAT CATCCGCAAT GGTGGTCTGG GCAATGGCGG CACCAACTTC GATGCCAAGA TCCGTCGTAA CTCCACCGAC CTTGAGGACC TCTTCATCGC TCACATCAGC GGTATGGATG CCATGGCCCG CGCTCTTGAG AACGCTGCCG CCATCCTGGA GGAGAGTGAA CTGCCCGCTA TGAAGAAGGA GCGCTATGCC AGCTTCGACA GCGGCATCGG CAAGGACTTC GAGGAAGGCA AACTCACCCT CGAGCAGGCT TATGAGTATG GCAAGAAAGT AGAAGAGCCG AAACAGATTT CTGGCAAGCA GGAGAAGTAC GAGACCATCG TCGCCCTGTA CTGCAAGTAA |
| SEQ 3: Natural xylose isomerase sequence | Met Ala Lys Glu Tyr Phe Pro Phe Thr Gly Lys Ile Pro Phe Glu Gly Lys Glu Ser Lys Asn Val Met Ala Phe His Tyr Tyr Asp Pro Glu Lys Val Val Met Gly Lys Lys Met Lys Asp Trp Leu Lys Phe Ala Met Ala Trp Trp His Thr Leu Gly Gly Ala Ser Ala Asp Gln Phe Gly Gly Gln Thr Arg Ser Tyr Glu Trp Asp Lys Ala Glu Asp Ala Val Gln Arg Ala Lys Asp Lys Met Asp Ala Gly Phe Glu Ile Met Asp Lys Leu Gly Ile Glu Tyr Phe Cys Phe His Asp Val Asp Leu Val Glu Glu Gly Ala Thr Ile Ala Glu Tyr Glu Glu Arg Met Lys Ala Ile Thr Asp Tyr Ala Gln Glu Lys Met Lys Gln Phe Pro Asn Ile Lys Leu Leu Trp Gly Thr Ala Asn Val Phe Gly Asn Lys Arg Tyr Ala Asn Gly Ala Ser Thr Asn Pro Asp Phe Asp Val Val Ala Arg Ala Ile Val Gln Ile Lys Asn Ala Ile Asp Ala Thr Ile Lys Leu Gly Gly Thr Asn Tyr Val Phe Trp Gly Gly Arg Glu Gly Tyr Met Ser Leu Leu Asn Thr Asp Gln Lys Arg Glu Lys Glu His Met Ala Thr Met Leu Thr Met Ala Arg Asp Tyr Ala Arg Ala Lys Gly Phe Lys Gly Thr Phe Leu Ile Glu Pro Lys Pro Met Glu Pro Ser Lys His Gln Tyr Asp Val Asp Thr Glu Thr Val Ile Gly Phe Leu Arg Ala His Gly Leu Asp Lys Asp Phe Lys Val Asn Ile Glu Val Asn His Ala Thr Leu Ala Gly His Thr Phe Glu His Glu Leu Ala Cys Ala Val Asp Ala Gly Met Leu Gly Ser Ile Asp Ala Asn Arg Gly Asp Ala Gln Asn Gly Trp Asp Thr Asp Gln Phe Pro Ile Asp Asn Phe Glu Leu Thr Gln Ala Met Leu Glu Ile Ile Arg Asn Gly Gly Leu Gly Asn Gly Gly Thr Asn Phe Asp Ala Lys Ile Arg Arg Asn Ser Thr Asp Leu Glu Asp Leu Phe Ile Ala His Ile Ser Gly Met Asp Ala Met Ala Arg Ala Leu Glu Asn Ala Ala Ala Ile Leu Glu Glu Ser Glu Leu Pro Ala Met Lys Lys Glu Arg Tyr Ala Ser Phe Asp Ser Gly Ile Gly Lys Asp Phe Glu Glu Gly Lys Leu Thr Leu Glu Gln Ala Tyr Glu Tyr Gly Lys Lys Val Glu Glu Pro Lys Gln Ile Ser Gly Lys Gln Glu Lys Tyr Glu Thr Ile Val Ala Leu Tyr Cys Lys |
| SEQ 4: Natural xylose isomerase gene sequence | ATGGCAAAAG AATATTTTCC GTTTACTGGT AAAATTCCTT TCGAGGGAAA GGATAGTAAA AATGTAATGG CTTTCCATTA TTACGAGCCC GAGAAAGTCG TGATGGGAAA GAAGATGAAG GACTGGCTGA AGTTCGCAAT GGCCTGGTGG CACACACTGG GAGGCGCTTC TGCAGACCAG TTCGGTGGTC AAACTCGCAG CTATGAGTGG GACAAGGCTG AATGCCCCGT ACAGCGTGCA AAGGATAAGA TGGACGCTGG TTTCGAGATC ATGGATAAGC TGGGTATCGA GTACTTCTGC TTCCACGATG TAGACCTCGT TGAGGAGGCT CCCACCATCG CTGAGTACGA GGAGCGCATG AAGGCCATCA CCGACTACGC TCAGGAGAAG ATGAAGCAGT TCCCCAATAT CAAGCTGCTC TGGGGTACCG CAAACGTATT CGGCAACAAG CGTTATGCCA ATGGCGCTTC TACCAACCCC GATTTCGATG TGGTTGCTCG TGCGATTGTT CAGATCAAGA ACTCTATCGA CGCTACCATC AAGCTTGGTG GTACCAACTA TGTGTTCTGG GGTGGTCGTG AGGGCTACAT GAGCCTGTTG AACACCGACC AGAAGCGTGA GAAGGAGCAC ATGGCTACGA TGCTGGGTAT GGCTCGTGAC TATGCTCGCG CTAAGGGATT CAAGGGTACG TTCCTGATTG AGCCGAAGCC GATGGAGCCT TCAAAGCACC AGTATGATGT GGACACAGAG ACCGTGATTG GCTTCCTGAA GGCACATGGT CTGGATAAGG ACTTCAAGGT GAACATCGAG GTGAACCACG CTACATTGGC TGGTCACACC TTCGAGCACG AACTGGCTTG TGCTGTTGAC GCTGGTATGC TGGGTTCTAT CGACGCTAAC CGCGGTGATG CCCAGAACGG CTGGGATACC GACCAGTTCC CCATCGACAA CTTTGAGCTG ACACAGGCTA TGCTGGAGAT CATCCGCAAC GGTGGTCTGG GCAATGGCGG TACCAATTTC GACGCCAAGA TCCGTCGTAA TTCTACCGAC CTCGAGGATC TCTTCATCGC TCATATCAGC GGTATGGATG CCATGGCCCG CGCCCTGATG AATGCAGCCG ATATTCTTGA GAACTCTGAA CTGCCCGCAA TGAAGAAGGC TCGCTACGCA AGCTTCGACA GCGGTATCGG TAAGGACTTC GAGGATGGCA AGCTGACCTT CGAGCAGGTT TACGAGTATG GTAAGAAGGT TGAAGAGCCG AAGCAGACCT CTGGCAAGCA GGAGAAGTAC GAGACAATCG TCGCCCTCCA CTGCAAATAA |
